# Aid effectiveness and programmatic effectiveness: a proposed framework for comparative evaluation of different aid interventions in a particular health system

**DOI:** 10.1186/s41256-017-0029-8

**Published:** 2017-04-03

**Authors:** Hasibul Haque, Philip C. Hill, Robin Gauld

**Affiliations:** 1 Department of Preventive and Social Medicine, Dunedin School of Medicine, University of Otago, Dunedin, 9016 New Zealand; 2 Centre for International Health, University of Otago, Dunedin, 9016 New Zealand; 3 Dean’s Office, School of Business, University of Otago, Dunedin, 9016 New Zealand

**Keywords:** Paris principles, Aid effectiveness, Impact evaluation, Development effectiveness, Health systems strengthening, Fragile states, Realist evaluation

## Abstract

**Background:**

Against a backdrop of changing concepts of aid effectiveness, development effectiveness, health systems strengthening, and increasing emphasis on impact evaluation, this article proposes a theory-driven impact evaluation framework to gauge the effect of aid effectiveness principles on programmatic outcomes of different aid funded programs in the health sector of a particular country.

**Methods:**

The foundation and step-by-step process of implementing the framework are described.

**Results:**

With empirical evidence from the field, the steps involve analysis of context, program designs, implementation mechanisms, outcomes, synthesis, and interpretation of findings through the programs’ underlying program theories and interactions with the state context and health system.

**Conclusions:**

The framework can be useful for comparatively evaluating different aid interventions both in fragile and non-fragile state contexts.

## Background

Discourses around more effective ways of achieving intended results and impacts of aid interventions are ongoing. Formulated around five central pillars: country ownership, alignment, harmonization, managing for results, and mutual accountability, the Paris Declaration on Aid Effectiveness [39] was endorsed to base development efforts on first-hand experience of what works and does not work with aid [35]. However, frameworks and country specific studies have been limited so far for evaluating the effect of these principles on programmatic outcomes of aid interventions in the health sector.

The need for such a framework is more acutely felt in fragile state contexts. Fragile states, as defined by their weak capacity, unresponsiveness, or lack of legitimacy to provide services to their people [18] are often worse off than the non-fragile states in terms of key health indicators and social determinants of health [19, 34, 46]. Due to the “problematic partnerships”, and often lack of basic institutions, policies, and adequate country systems related to financial management, procurement, and monitoring and evaluation to which donors can align their efforts, the applicability of the Paris principles in fragile and conflict affected situations are often found challenging [27, 37].

Using program theory and a realist evaluation approach, this article presents a framework to evaluate the downstream effect of adherence to the Paris principles on programmatic effectiveness of different aid interventions in a particular health sector. This framework was pilot tested in a fragile state’s context by comparatively evaluating three externally funded programs in Timor-Leste’s health sector [24]. These programs were the AusAID and World Bank funded Health Sector Strategic Plan Strengthening Project (HSSP-SP) that followed a sector wide approach (SWAp); the Global Fund funded National HIV/AIDS and STI Program (NAP) that used a government mechanism but different financial management and monitoring systems; and the USAID funded Immunizasaun Proteje Labarik (IPL) that used an NGO contracting mechanism. Based on the pilot, this article describes step by step methods of using the framework, and discusses the foundation and feasibility of using this approach for comparative evaluation of different aid funded programs both in fragile and non-fragile states’ health sector context.

### Related literature

The field of aid effectiveness evaluation has been evolving: there has been a shift from an initial focus on the effect of aid on poverty alleviation to efficiency and transparency of aid management processes and, more recently, to the impact of aid funded programs [5, 6, 33, 45, 54]. While the Paris Declaration [39] focused on aid delivery policy, instruments, design and operations of aid programs, the Fourth High Level Forum on Aid Effectiveness held in Busan further attempted to link the Paris principles of aid effectiveness more explicitly to programmatic effectiveness, and in a broader sense, to development effectiveness [7].

The Organisation for Economic Co-operation and Development (OECD) undertook a series of surveys and an independent evaluation of the implementation of the Paris principles at the country level by employing a theory-driven evaluation approach. Although these studies claim a high level “growing evidence” of plausible effect of the implementation of aid effectiveness principles on better health outcomes, “in broad terms” [58, 59], they lack focus on the specific country contexts and evaluation of particular aid interventions. The International Health Partnership (IHP+), on the other hand, developed a common monitoring and evaluation (M&E) framework that link the input and processes (such as governance, financing and implementation context) to the outputs, outcomes, and impact of a particular invention or the health sector of a country as a whole [57]. The Scaling Up Nutrition Movement also developed a monitoring and evaluation framework to document changes related to the impacts, outcomes, and outputs of scaling up nutrition program at the country level and to link them to the contributing activities and stakeholders to measure the progress and contributions of different players against set targets [51]. In a similar fashion, based on the logical chain between implementation process, health system strengthening, and health outcomes in the context of monitoring results of aid effectiveness in the health sector, Paul et al. [42] conducted a three-level assessment in Mali for the process, systems effect, and health outcomes. Although these studies and frameworks provided feasible approaches to aid effectiveness evaluation, they were not applied to comparative evaluation of different aid intervention approaches in a particular context, and thus missed the opportunity for further investigation for establishing a causal relation between adherence to the Paris principles and programmatic effectiveness.

### Proposed framework for comparative evaluation of aid interventions

The OECD-DAC [36] summarized the aim of evaluation as “to determine the relevance and fulfilment of objectives, development efficiency, effectiveness, impact and sustainability” of an on-going or completed project, program or policy. However, as pointed out by Stufflebeam et al. [49], there are numerous different approaches that informed and shaped the evolving practice of evaluation. Approaches to evaluation that fall within the “positivist paradigm” focus on methodological rigor and advocate for a traditional scientific approach with quasi-experimental research designs, use of counterfactual measurement, and validity and reliability of findings [1]. Evaluation models that follow a constructivist approach, on the other hand, attempt to interpret reality from the ‘voice of stakeholders’ by adopting participatory methods, case-studies and observations [15]. While the quasi-experimental study designs are criticized for ignoring “context-sensitive” information [26] and for their apparent inability to provide valid findings when applied to a dynamic and complex system [50], the traditional case-study methods are also challenged with “low external validity and low power to explain change” [10, 14].

Recognizing the need for a different evaluation approach for innovative programs in a complex and dynamic environment, Patton [41] proposed a utilization oriented evaluation approach known as developmental evaluation. Grounded in systems thinking and responsive to the context, developmental evaluation allows for methodological flexibility, adaptability, tolerance to ambiguity, and use of creative and critical thinking to conduct the evaluation as an integrated part of the intervention itself [40].

Broadly referred to as theory-driven evaluation [11] and “realist evaluation”, another similar approach assesses not only the implementation of a program and its effectiveness, but also the causal mechanisms and contextual factors that underlie outcomes by mapping out the causal chain between the inputs, outputs, outcome, and impact [22, 47] to provide more detailed context-sensitive information for decision makers and to indicate ‘what works, how, in which conditions and for whom’ [43]. As explained by de Savigny and Adams [48], direct and indirect results of health programs are influenced by their interactions with the context and health systems. But, at the same time, it is argued that the context is not purely an external factor, as the context is also shaped by the interventions and their activities [3]. Program outcomes in a fragile state’s health sector are, therefore, more sensitive to the programs’ interactions with state fragility and health system context making it more appropriate to employ a realist evaluation than any other approach to analyze the interaction between the context and mechanism for evaluation of the outcomes.

**Fig. 1:**
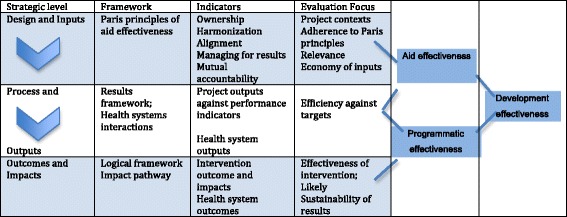
Theoretical framework for aid effectiveness evaluation. Source: Authors

For an aid effectiveness evaluation, we argue that the ‘mechanisms’ (or how the aid interventions are implemented and interact with the context) are shaped by agreed policies such as the Paris principles, while the health system provides the context. Therefore, the Paris principles and health systems thinking fit into a theory-driven realist evaluation framework for evaluating the aid interventions, as illustrated in Fig. 1.

This framework assumes that adherence to the Paris principles works at the program design and implementation process level, contributing to greater sense of ownership, alignment, harmonization, and policy coordination along with an emphasis on results and accountability. This, in turn, contributes to reduced transaction costs, increased efficiency, and increased efforts in health systems strengthening. Outputs from greater adherence to the Paris principles and programmatic results then contribute to increased effectiveness of achieving program objectives, increased health system strengthening outcomes, and, ultimately to the sustainable impact of the program. The country and health system’s context plays an active role throughout this chain with possible interactions with and effects on the inputs, processes, and outcomes.

## Methods

As mentioned earlier, the above framework was applied to compare and evaluate three different aid interventions from Timor-Leste’s health sector through the following steps:

1. Analyze the context of state fragility and health system and identify the possible drivers and barriers that may be shaped by and influence the program outcomes;
2. Analyze the program designs and mechanisms and see to what extent the aid interventions adhered to the Paris principles and how they interacted with the state fragility and health system;
3. Conduct an appraisal of program outcomes according to their objectives, underlying program theories, and intended or unintended effect on the health system;
4. Evaluate the aid interventions by comparing to what extent they adhered to the Paris principles, their interactions with the state fragility and health system context, and outcomes related to the program objectives and health system to see their relative effectiveness in comparison to each other and to infer if there is any causal relationship between an intervention’s adherence to the Paris principles and its programmatic effectiveness (Table 1).

**Table 1:** Summary of evaluation design and methods

Step	Method/approach	Analysis and expected outcome
Step 1: Context analysis: Analyze country context, state fragility, and health system contexts of the aid interventions under evaluation	Qualitative approach through realist synthesis methods	Identify drivers and barriers from state fragility and health system that can be shaped by the interventions and can influence intervention outcomes
Step 2: Program design and mechanism analysis: Analyze program designs, elicit underlying program theories, and identify how aid interventions interact with the state fragility and health system context	Qualitative-interpretivist approach and constructivist-mixed methods approach through document review, semi-structured interviews of stakeholders; focus group discussions	Using both qualitative and quantitative analysis, elicit underlying program theories of each intervention and their implementation mechanisms. Construct stakeholders’ views on the extent each intervention adhered to different aspects of the Paris principles
Step 3: Outcome analysis: Analyze program outcomes and their values in terms of achievement of targeted objectives and effect on health system	Flexible methods depending on type and purpose of an intervention. A suggested example is: quantitative analysis of cost-effectiveness of outcomes using epidemiological modeling exercise with actual, counterfactual and optimum scenario modeling	Analysis of degree of achievement of targeted outcomes, cost effectiveness and technical efficiency of each program
Step 4: Comparative evaluation of inputs, process, and outcomes	Realist synthesis with comparative analysis of relevance and adherence to the Paris principles, efficiency, effectiveness, and likely sustainability of each intervention	Interpret significance of findings by comparing them with program theories, empirical evidences from qualitative and quantitative analysis, and plausible context-mechanism-outcome interactions. Investigate possible causal chain between adherence to the Paris principles and programmatic effectiveness

### Step 1: context analysis

Context analysis attempted to draw key considerations of a program’s setting including the country’s geography, history, culture, economy, politics, human development status, state fragility, and health systems. A range of analytical tools including mapping actors [9], force-field analysis [16], SWOT (strengths, weaknesses, opportunities and threats) analysis [53], and PEST (political, economic, social and technological environment) analysis [17] were used for this purpose.

The six building blocks of the health systems: governance and leadership, health service delivery, health information, health work-force, health commodities and technology, and health financing [56] were analyzed by using available published work and documents from the government and development partner sources. The structure and organization of the health system were then compared with the state fragility context and health system’s performance to identify the drivers and barriers for the programs and any possible effects of the context on the program performance.

### Step 2: program design and mechanism analysis

At this stage, program related documents including program proposals, program agreements, budget, work plan, performance frameworks, progress reports and assessments were reviewed to elicit the underlying program theories, and assess their identified needs, planned activities, programmatic mechanisms, relevance to their objectives, results framework, and possible impact pathways in their interactions with the health system and state fragility.

**Fig. 2:**
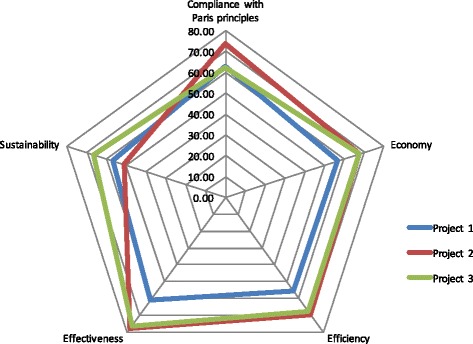
Scores derived from stakeholder opinions on different aspects of three aid-funded programs

In order to understand the program ‘mechanisms’ [43], a constructivist approach [23] was used by involving the stakeholders to construct an understanding of how the interventions worked. This was done by developing an assessment questionnaire and then conducting interviews and focus group discussions with sample stakeholders for collecting their views on the design, process, results, efficiency, effectiveness, and likely sustainability of the program results in comparison with the best practices. Information obtained from interviews, filled in evaluation questionnaires, and focus group discussions were analyzed to derive a composite score for each intervention on the aspects of their adherence to the Paris principles, economy, efficiency, effectiveness, and likely sustainability. These scores were then presented in the form of a “balanced scorecard” [25], as shown in Fig. 2.

### Step 3: outcome analysis

For each intervention, outcomes related to both program objectives and health system were measured based on a flexible approach according to the nature of the program. A cost effectiveness analysis was attempted to calculate the cost per different outcome measures by each program. In the pilot, this was done by measuring the outcomes of a program and comparing them with the likely outcomes from two other statistically modeled scenarios: 1) the null or control scenario without the program; and 2) the optimal scenario if all the program targets were achieved and all the allocated resources were fully utilized. In order to make the cost-effectiveness analysis of each program comparable to each other, technical efficiency [21] of each program was calculated by comparing the cost per an outcome measure achieved by the program with the likely cost per the same outcome measure by the program in the optimal scenario.

### Step 4: comparative evaluation of aid interventions

This step conducted evaluation per se of each intervention in terms of their relevance, efficiency, effectiveness and likely sustainability and compared these aspects of each program with the same aspects of other programs under evaluation along with their extent of adherence to the Paris principles. A triangulation, synthesis, and further investigation were then conducted to see if there was any causal chain between the adherence to the Paris principles and programmatic effectiveness.

## Results and discussion

As described in Steps 1–4, the pilot used mixed methods [20] with number of analytical tools for country context, state fragility, and health system analysis; a balanced scorecards approach for collecting and analyzing stakeholder perceptions; cost-effectiveness and technical efficiency analysis for program outcomes; data envelopment analysis (DEA) [2] for comparative efficiency analysis; and a correlations analysis for possible association between adherence to the Paris principles of aid effectiveness and programmatic effectiveness. Figure 2 provides an example of comparative analysis of three aid-funded programs as evaluated by the stakeholders.

In order to measure and compare multiple health outcomes of the programs, the pilot used disability adjusted life year (DALY) that provided a single health index based on number of deaths and new episodes of disease averted and improvement of quality of life achieved [32]. A quasi-experimental design was used for cost-effectiveness analysis by mathematically modeling the counterfactual scenarios of likely effect of absence of a program on outcomes and likely effect on outcomes for the optimal scenario with 100% achievement of targets. Results were then validated by comparing and triangulating findings from different techniques in relation to their underlying program theories. While this pilot exposed highly useful information for comparative evaluation of different aspects of the aid interventions, the correlation analysis for their adherence to the Paris principles and programmatic effectiveness could not be concluded due to a small sample size.

As extensively reviewed by Coryn et al. [13] and Marchal et al. [31], realist evaluation has been increasingly applied in a variety of fields within health systems research. It is argued that realist evaluation opens the “black box” and provides a useful framework to examine how context and mechanisms influence the outcomes of a program [4, 12, 22, 38, 52]; and this approach is well suited to investigating complexity, either for evaluations of complex programs [8, 29, 30, 44, 55] or of complex causal pathways [47]. It is, therefore, believed that realist evaluation offers an opportunity to develop an “integrated outcome and process evaluation framework” [28]. However, in practice, realist evaluation still suffers from a number of challenges including: lack of methodological guidance, lack of consensus on the definition of ‘mechanism’, difficulties to differentiate mechanism from context, and difficulties to apply the principles of realist evaluation in practice [31].

This pilot identified a few strengths of the proposed framework for realist evaluation. First, the framework allows for consideration of state fragility, health system related issues, and any policy guidelines such as the Paris principles of aid effectiveness in the evaluation. At the same time, it can evaluate the policy guidelines in terms of their effect on programmatic outcomes. Second, the framework provides flexibility to choose from a range of qualitative and quantitative tools and techniques from different disciplines to suit to the analytical needs of selected programs. Third, the framework addresses the challenge of defining mechanisms in a complex situation such as that of a fragile state by having the stakeholders assessing the selected programs by comparing with the best practices, and, in comparison with each other. This “constructivist” assessment of the programs by the stakeholders actually indicates the mechanism of the programs, as Greenhalgh et al. [22] defined mechanisms as “the stakeholders’ ideas” about how changes are achieved. Fourth, use of this framework for comparative evaluation within the same context also addresses the challenge of separating mechanism from the context. Based on the “context-mechanism-outcome” configuration of realist evaluation [43], it is assumed that in a given context (such as that of a fragile state’s health sector), different mechanisms, by interacting with the same context, would result in different outcomes. Therefore, in a given context, the performance of different aid interventions (or the mechanisms) can be fairly compared with each other by exploring their interactions with the context and by comparing their efficiency of achieving outcomes based on the evaluation criteria and without needing to separate the mechanisms from the context.

However, the challenges for putting the proposed framework in practice may include: lack of required data; difficulty to measure outcomes of some programs, for example, for a health system strengthening program; dealing with contribution issues and confounding factors that may affect outcomes; and time and cost associated with selecting a feasible sample of programs for comparison. Despite these common challenges that can be applicable for any evaluation designs, the proposed framework presents a feasible approach for comparatively evaluating different aid interventions at the national level in a complex and dynamic context.

## Conclusion

The framework presented in this article provides a generic conceptual model, which can be used not only in a fragile state’s setting, but also for evaluating a number of heterogeneous interventions from any particular setting to investigate aid effectiveness, programmatic effectiveness, and interplay of these two constructs. Application of this framework in large-scale evaluative research can further contribute to the shaping up of the concepts of aid effectiveness, development effectiveness, impact evaluation, health system strengthening, and their possible interplay.

## Abbreviations

DALY: Disability adjusted life year
DEA: Data envelopment analysis
HSSP-SP: Health sector strategic plan support project (in Timor-Leste)
IHP+: International health partnership
IPL: Immunizasaun Proteje Labarik (Children Immunization Project in Timor-Leste)
M&E: Monitoring and evaluation
NAP: National HIV/AIDS and STI Programme (in Timor-Leste)
OECD: The organisation for economic co-operation and development
PEST: Political, economic, social and technological environment
SWOT: Strengths, weaknesses, opportunities and threats
WHO: World health organization
